# Fear-Avoidance Beliefs Are Associated with Pain Intensity and Shoulder Disability in Adults with Chronic Shoulder Pain: A Cross-Sectional Study

**DOI:** 10.3390/jcm12103376

**Published:** 2023-05-10

**Authors:** Joaquín González Aroca, Álvaro Puelles Díaz, Carlos Navarrete, Loreto Albarnez

**Affiliations:** 1 School of Kinesiology, University of La Serena, La Serena 1700000, Chile; 2 Department of Mathematics, Faculty of Science, University of La Serena, La Serena 1700000, Chile

**Keywords:** fear, pain measurement, chronic pan, disability evaluation, psychometrics, analysis of variance

## Abstract

Shoulder pain is one of the most common musculoskeletal conditions, and for people over 40 years old, it represents the musculoskeletal pain with the greatest impact on quality of life. Psychological factors, such as fear-avoidance beliefs, are associated with musculoskeletal pain, and several studies suggest that they can influence various treatment outcomes. Our objective was to explore the cross-sectional association between fear-avoidance beliefs and shoulder pain intensity and disability in subjects with chronic shoulder pain. A cross-sectional study was conducted, and 208 participants with chronic unilateral subacromial shoulder pain were recruited. The shoulder pain and disability index assessed pain intensity and disability. The Spanish fear-avoidance components scale assessed the presence of fear-avoidance beliefs. The association between fear-avoidance beliefs and pain intensity and disability was analyzed by means of multiple linear regression models and proportional odds models, reporting odds ratios and 95% confidence intervals. Shoulder and pain disability scores were significantly associated with fear-avoidance beliefs (*p* < 0.0001, adjusted R-square 0.93, multiple linear regression). There was no evidence of an association between sex and age in this study. The regression coefficient for shoulder pain intensity and disability score was 0.67446. The proportional odds model showed an odds ratio of 1.39 (1.29–1.50) for shoulder pain intensity and disability total score. This study suggests that greater levels of fear-avoidance beliefs are associated with greater levels of shoulder pain and disability in adults with chronic shoulder pain.

## 1. Introduction

A pain complaint can be considered chronic if it persists beyond the normal healing time for a particular injury or tissue damage. Typically, pain lasting longer than three months is considered chronic, and the cause of pain can be due to ongoing pathology or it can persist even after the tissue has healed. Chronic pain is a standalone medical condition that involves persistent pain and has components that include psychological, neurologic, social, and physiological factors [1]. In the context of chronic musculoskeletal pain, managing symptoms can be challenging and is associated with five factors: age, self-management support, support from non-healthcare providers, religion or spirituality, and overall health [2].

Shoulder pain is a prevalent musculoskeletal condition with a multifactorial etiology and is associated with several disorders, including diabetes, high blood pressure, psychological factors, thyroid dysfunction, and obesity [3]. The prevalence of shoulder pain ranges from 6.9% to 26% in the general population [4], with a lifetime prevalence of up to 67% [5]. It generates alterations in work capacity and psychological stress [6,7], and for people over 40 years old, it represents musculoskeletal pain with the greatest impact on quality of life [8].

Only half of the people who suffer a new episode of shoulder pain manage to fully recover within the first 6 months, while 40% have persistent pain for more than 1 year [9]. This could be explained by the fact that the treatments focus mainly on the damaged tissue [10]. However, the pain may persist once the tissue damage has resolved, and pathological diagnosis together with imaging does not correlate with the intensity of shoulder pain [11,12,13,14].

Psychological factors have been investigated to further understand the experience of people with musculoskeletal pain. These factors have been found to be associated with shoulder pain and disability [15] and may also influence various treatment outcomes [16]. From this perspective, fear is a significant factor that can be defined as an emotional response to a recognizable and imminent danger, prompting specific physiological, cognitive, and behavioral reactions to confront the threat [17]. Fear learning serves as an adaptive survival mechanism that helps to identify actual or potential threatening signals, allowing for the initiation of appropriate defensive behaviors (e.g., escaping) to prevent harm [18]. Thus, to predict physical damage, people learn to fear movements and activities that they experience as painful [19], and as a consequence, they can adopt different behaviors, such as avoiding the feared movements/activities, in order to control further damage to the body [20]. Despite their protective effect when pain is acute, pain-related fear and avoidance may be detrimental in chronic pain [21].

Fear responses can emerge prior to the actual performance of movements that are expected to be painful [22]. This phenomenon is attributed to the activation of memory representations of the movement–pain association, triggered by the mere imagination of the painful movement [23]. Further evidence supports the acquisition of pain-related fear through non-direct experience, including observation, instruction, the symbolic representation of pain, and derived relationships such as conceptual equivalence between stimuli [24,25], and to explain this, the fear-avoidance model, which has been extensively studied, when used to explain musculoskeletal pain, suggests that people with fearful and catastrophic thinking traits are more likely to develop chronic pain. The model describes how people who perceive pain as a threat exhibit protective behaviors in order to prevent further injury [26,27]. These behaviors are modifiable in the acute stage of pain [28]. However, when pain persists for a long time, modifying these behaviors is very complex. This inadequate management of pain leads to disuse, causing physical and psychological consequences that generate more pain and disability [29].

The fear-avoidance model of chronic musculoskeletal pain considers fear and avoidance related to pain as crucial factors in the transition from acute to chronic pain [30]. In subjects with low back pain, fear-avoidance beliefs have been identified as a risk factor in the development of chronic pain [31]. However, in the population with shoulder pain, its association with fear-avoidance beliefs remains unclear. In a systematic review conducted by Martínez-Calderón et al. [32], the results suggest that higher levels of fear avoidance and kinesiophobia at baseline predicted greater disability during the SP course; however, the included studies were assessed at high risk of bias and the certainty of the evidence was very low.

In regard to the clinical course of shoulder pain, the fear-avoidance beliefs of individuals receiving conservative treatments, such as physical therapy, have no predictive value on the severity of pain or disability, and these outcomes seem to depend on the kind of treatment received. After surgery, initial fear-avoidance beliefs can predict the result, but with physiotherapy, there is no such prediction regarding fear-avoidance beliefs. This distinction highlights the chance for physiotherapy to address negative beliefs early on to improve treatment outcomes [33].

Although it has been possible to determine prognostic factors associated with the chronicity of shoulder pain such as a high score on the shoulder pain and disability index (SPADI) and pain persistence for more than three months, the causes for the pain becoming chronic is uncertain [34]. The aim of this study was to explore the association and between pain intensity and shoulder disability with fear-avoidance beliefs (FAB) in individuals with chronic pain.

## 2. Materials and Methods

### 2.1. Study Design and Ethical Aspects

This cross-sectional study was conducted according to the Declaration of Helsinki and the STROBE statement [35]. This study was approved by the Ethics Committee of Universidad Santo Tomás (92/21).

### 2.2. Participants and Setting

Convenience sampling was carried out, and 208 subjects with chronic unilateral subacromial shoulder pain in their dominant extremity were recruited from May 2021 to April 2022 in a private clinic located in the city of Coquimbo, Chile. Recruitment was carried out by a general practitioner; therefore, each subject with shoulder pain was referred to the physiotherapy service. In this way, 386 potentially eligible people were gathered, of which 100 were excluded for not meeting the inclusion and exclusion criteria. The 286 participants who satisfied the inclusion criteria were invited to participate and provided written informed consent. A total of 77 subjects refused to participate in the study. The sample size was determined for convenience. All assessments were performed by a physiotherapist trained in musculoskeletal rehabilitation.

The inclusion criteria were (1) at least 18 years old; (2) Chilean nationality; (3) chronic unilateral shoulder pain (pain duration more than 3 months) in the dominant extremity; (4) pain located in the anterior and/or lateral region of the shoulder; (5) the presence of a painful arc in abduction–flexion movements; (6) a positive Neer’s sign or Hawkins–Kennedy test; and (7) the presence of pain when performing humeral external rotation against resistance or humeral abduction, or a positive Jobe Test [36].

The exclusion criteria were as follows: (1) history of significant shoulder injury (e.g., humeral head fracture or massive rotator cuff tear); (2) diagnosis of frozen shoulder characterized by the loss of passive and active range of motion, mainly in external rotation [37]; (3) previous shoulder surgery; (4) previous diagnosis of cancer; (5) injection of corticosteroids in the last 2 months; (6) origin of pain in the cervical spine determined by the reproduction of symptoms in physiological movements of this segment and a positive spur test; and (7) the presence of severe shoulder osteoarthritis, previous cerebrovascular accident, or rheumatoid arthritis.

### 2.3. Outcome Measures

#### 2.3.1. Fear-Avoidance Beliefs

To assess fear-avoidance beliefs, the Spanish fear-avoidance components scale (FACS) was used. This is a comprehensive measure of fear avoidance based on the well-established chronic pain fear-avoidance model. It consists of two factors—the general avoidance of fear, which contains 14 items, and the type of activities that are avoided, which contains 6 items—and each item presents a score from zero (totally disagree) to 10 (totally agree) with a total possible score of 100. Five severity levels are available for clinical interpretation: subclinical (0–20), mild (21–40), moderate (41–60), severe (61–80), and extreme (81–100) [38]. The FACS in the shoulder pain population has good reliability (ICC between 0.75 and 0.90) and adequate internal consistency (Cronbach’s alpha > 0.70) [39].

#### 2.3.2. Shoulder Pain Intensity and Disability

The shoulder pain and disability index (SPADI), Spanish version, assesses the intensity of shoulder pain and disability. This tool is made up of 13 items divided into two subdomains—pain intensity and disability—and each item has a categorized score from zero (no pain or difficulty) to 10 (maximum pain or difficulty). The score in the pain subdomain has a range from 0 to 50, while disability has a range from 0 to 80; this results in a total scale range of 0–100. Higher scores indicate higher levels of disability and pain intensity. This scale presents good psychometric properties to assess pain intensity and disability (intraclass correlation coefficient = 0.992) [40].

### 2.4. Statistical Analysis

The association between FACS (the response) and SPADI scores was analyzed by means of multiple linear regression models and proportional odds models. Proportional odds models are a simplified and more parsimonious case of the more general multinomial logistic regression models. Likelihood ratio tests were used to determine that the simpler model was indeed adequate for the data. Multinomial logistic models are a special case of generalized multiple linear models where the probability of classifying a sample unit in one of *J* categories is modeled by means of cumulative logistic transformation.

$$\log\left(\frac{P(Y\geq j)}{P(Y<j)}\right)=\log\left(\frac{P(Y\geq j)}{1-P(Y\geq j)}\right)=\log\left(\frac{\pi_{j+1}+\ldots +\pi_{J}}{\pi_{1}+\ldots +\pi_{j}}\right)$$

for *j* = 1, 2,…, *J*. In our case, we have *J* = 3 groups defining the moderate, severe, and extreme classification in the FACS. This transformed response is modeled as a linear regression, where the exponential of the coefficients defines the odds ratio of the higher groups compared to the lower groups. All calculations were performed using R statistical software (4.0.2, Vienna, Austria). Results are presented as mean (S.D.) or proportion (percentage) where applicable. Unadjusted associations between continuous variables were reported by means of Pearson correlations. A *p* value less than 0.05 was used to determine significance.

## 3. Results

### 3.1. Sample Characteristics

Of the total sample, 47.1% were male. A total of 45.7% of the subjects had extreme FAB, 33.2% severe, and 13.5% moderate. The age frequency of the sample according to tertiles was 35–44 = 61 subjects; 45–52 = 76 subjects; and 53–59 years = 71 subjects. Table 1 shows the characteristics of the sample.

### 3.2. Relation between Age, Fear-Avoidance Beliefs, and Shoulder Pain Intensity and Disability

Correlations between age, fear-avoidance beliefs, and shoulder pain intensity and disability data are reported in Table 2.

### 3.3. The Association between Fear-Avoidance Beliefs and Shoulder Pain Intensity and Disability

**Figure 1:**
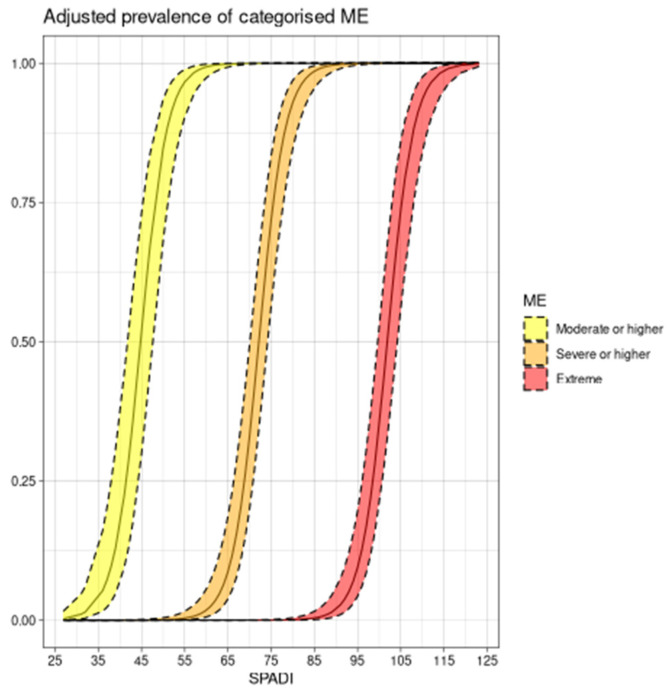
Proportional odds model shown in probability scale for SPADI total score; ME, fear-avoidance component scale; SPADI, shoulder pain and disability index total score.

The SPADI score showed a significant association with FACS (*p* < 0.0001, adjusted R-square 0.93, multiple linear regression). There was no evidence of an association between sex and age in this study. The regression coefficient for SPADI was 0.67446, meaning that a 10-point increase in the SPADI score corresponds to an average increase of 6.7 points in the FACS. We further studied FACS categorized as moderate, severe, and extreme and its association with the SPADI score. The proportional odds model showed an odds ratio of 1.39 (1.29–1.50) for the SPADI score, meaning roughly a 39% average increase in the odds of being classified in the upper-next ME category for one incremental unit in the SPADI score (*p* < 0.0001). We show the results in a probability scale in Figure 1. In that figure, it can be seen that, for instance, a 50% prevalence of moderate or higher FAB is attained at roughly 45 points on the SPADI, 70 points on the SPADI for severe or higher FAB, and 100 points for extreme FAB.

## 4. Discussion

The purpose of this study was to explore the cross-sectional association between fear-avoidance beliefs and both pain intensity and shoulder disability in subjects with chronic shoulder pain.

This study showed that higher levels of shoulder pain intensity and disability were associated with higher levels of fear avoidance. These shoulder pain intensity and disability results contributed to explain 93% of the variance in the total FACS score, and we found an odds ratio of 1.39 (1.29–1.50) for the SPADI score, meaning roughly a 39% average increase in the odds of being classified in the upper-next FAB category for one incremental unit in the SPADI score.

The proportional odds model shows an association between shoulder pain intensity and disability and fear-avoidance severity. We propose to use this graph (Figure 1) in the planning of treatment goals focused on addressing pain beliefs. In this way, a decrease in the SPADI score in relation to FAB severity could be estimated.

Pain serves as a protective mechanism after an injury to avoid further harm. This includes decreased mobility in the affected area and the onset of cognitive and emotional responses, such as fear of movement, which may stem from prior experiences and psychological distress. This creates a cycle where negative thoughts decrease the ability to cope with pain. Evidence shows that individuals with chronic musculoskeletal pain have structural and functional changes in their brains primarily in the mesolimbic and prefrontal regions [41]. These regions play a role in processing incoming stimuli and impacting the cognitive and behavioral aspects of pain. Impaired activity in these regions leads to hypervigilance to pain and a decreased ability to manage it. Notably, these regions communicate with the brainstem and sensorimotor centers, enabling neuroplastic changes and affecting descending pain modulation systems [42,43]. A crucial component of fear-avoidance behavior is the maladaptive search for safety, characterized by persistent hypervigilance. Given that the shoulder joint exhibits the greatest range of motion in the body [44], it is possible that our findings of elevated levels of fear avoidance could be explained by this biomechanical characteristic.

Regarding sex differences in pain response, our findings contrast with previous reports [45]. Studies have found that pain intensity in patients with chronic low back pain is related to pain anxiety in men and fear of injury in women [46]. Women have also been found to experience higher levels of depression related to back pain [47]. Pain catastrophizing plays a role in the differences in coping strategies for cold pressor pain between young adult males and females. Furthermore, gender role expectations have been found to significantly impact pain experiences, with those holding stronger beliefs about such expectations conforming more to their gender’s perceived role [48].

The fear-avoidance model has been studied mainly in subjects with low back pain. In this population, different studies have shown that higher levels of fear avoidance are associated with higher levels of pain, disability, and worse chronic pain prognosis [49,50]. In line with our results, a clinical trial conducted by Janela et al. [51] demonstrated that higher levels of fear-avoidance beliefs were associated with higher levels of disability in the upper extremities when applying a treatment based on physical exercise and psychoeducation. However, Riley et al. [52] found no association between shoulder pain intensity and disability and fear-avoidance beliefs in subjects with chronic shoulder pain, although this could be due to random error caused by a small sample size (*n* = 30).

In the context of traumatic shoulder injuries, Lemaster et al. investigated whether FAB is associated with shoulder function in a small traumatic rotator cuff tear sample with chronic pain. This study found that better shoulder function was associated with increased arm flexion, increased scapular rotation during arm flexion, and decreased fear-avoidance beliefs [53].

A recent study [54] exploring pain-related fear phenotypes found relevant results. The authors had two objectives: (1) to distinguish and compare the traits of pain-related fear phenotypes among individuals with shoulder pain and (2) to examine the relationship between demographic and clinical characteristics and self-reported upper limb function in individuals with shoulder pain. They conducted a cluster analysis using the variables of kinesiophobia, fear avoidance, and pain catastrophizing. They found two phenotype profiles associated with kinesiophobia, fear avoidance, pain catastrophizing, and upper limb function in individuals with shoulder pain. The results showed that individuals with negative behavioral cognitions such as fear of movement, fear avoidance, and catastrophic thoughts had worse self-reported function of the upper limbs and higher shoulder pain intensity and were older. This suggested that pain-related fear phenotypes were linked to self-reported upper limb function and the side affected. Their findings are in line with our results, since the mean age of the oldest age cluster (42.2 ± 13.8) is comparable to that in our sample (48.45 ± 6.05), and this could explain the association we observed.

On the other hand, a phenomenon closely related to fear avoidance is kinesiophobia, which is defined as “an extreme form of fear of movement, is defined as an excessive, irrational, and debilitating fear to execute a determined movement or activity owing to a feeling of vulnerability to a painful injury or reinjury” [55]. A few studies have explored the cross-sectional association between the SPADI and kinesiophobia, showing associations that explain between 19% and 31% of the total variance in shoulder pain and disability [56,57]. In an investigation conducted by Suer et al., they found that greater baseline kinesiophobia and pain catastrophizing were predictors of greater postoperative pain following nonarthroplasty shoulder surgery [58]. These findings support the idea that psychological factors should be assessed in the early stage of pain symptoms.

Several interventions have been shown to reduce FAB, including cognitive behavioral therapy, which is a psychological treatment with the goal of providing patients with strategies for coping with pain to reduce functional disability and typically involves combining various cognitive and behavioral strategies in its manuals to cater to the needs of a wider range of patients [59]. Another intervention is in vivo graded exposure, a therapeutic approach based on the fear-avoidance model aimed at reducing fear avoidance in people with chronic pain. The method is similar to the treatment of phobias and anxieties, where patients are exposed to fear-provoking stimuli. For those with chronic pain, the feared stimuli usually involve movements that are associated with pain or the possibility of back injury [60]. The efficacy of this exposure therapy has been studied through single case studies and randomized controlled trials. This treatment has often been compared to graded activity, a technique commonly used to reduce avoidance behaviors in cognitive behavioral therapy protocols [61]. Both of these interventions have been shown to be effective in chronic low back pain. Thus, considering the association that was shown in the present study, those interventions need to be studied in shoulder pain to see their effect.

A recent systematic review [62] has shown that exercise training may be an effective way to decrease fear-avoidance beliefs compared to other types of non-exercise training. Exercise training was found to be more effective in reducing fear-avoidance beliefs than a control group that received no intervention or waitlist control. However, when compared to non-exercise treatments such as cognitive behavioral therapy or education, exercise training alone was not found to be more effective in reducing fear-avoidance beliefs.

Future investigations should prospectively address psychological factors in the chronic shoulder pain population to verify their prognostic value.

Most of the studies that evaluate the prognostic factors and the effectiveness of interventions regarding pain beliefs are carried out in the low back pain population. Therefore, future research should study the effects of these factors on chronic shoulder pain.

### 4.1. Clinical Implications

The study emphasizes the significance of healthcare providers comprehending the patient’s views, prior experiences, and thoughts regarding their shoulder pain.

Additionally, it is crucial for them to be aware of their own biases and beliefs, as they can impact the patient’s fear. Our findings reinforce the importance of considering psychological aspects, specifically fear-avoidance beliefs, at the onset of pain. Other psychological elements, such as kinesiophobia, pain catastrophizing, and anxiety, play a role in chronic pain and must also be addressed. Addressing these psychological factors can not only prevent chronic pain but also create a positive therapeutic relationship with the patient through communication-based pain management interventions. The present study supports the utilization of the biopsychosocial model, which acknowledges both the physical and psychological aspects of pain. Healthcare providers must understand the impact of treatments such as exercise, not just physically but also on psychological well-being, resulting in enhanced patient confidence and self-efficacy and encouraging autonomous behaviors.

### 4.2. Methodological Considerations

The main limitation of this study is its cross-sectional design, with which we cannot establish temporal relationships. Regarding the participants, the sample was taken from a private clinic with a high flow of subjects who consulted for musculoskeletal pathologies; therefore, there is a risk of selection bias.

In our study, the measurement of the prevalence of shoulder pain and fear-avoidance beliefs could be affected by several factors such as the defined period for the analysis, evolution of the disease, and the complexity level of the private clinic where the sample was obtained. In this study, to measure shoulder pain intensity, shoulder disability, and fear-avoidance beliefs, the shoulder pain and disability index and fear-avoidance component scale were used, both validated in Spanish. However, none of them are validated yet for the Chilean population. We also understand that cases with long-lasting pain may have been overrepresented and those with short-lasting pain may have been underestimated. Finally, we are aware of the importance of conducting probabilistic sampling in cross-sectional studies. However, for technical reasons, it was impossible for our research group to carry out this type of sampling.

## 5. Conclusions

This study provided preliminary evidence about the association between fear-avoidance beliefs and chronic shoulder pain intensity and disability. Greater levels of fear-avoidance beliefs were associated with greater levels of shoulder pain and disability.

## Figures and Tables

**Table 1:** Characteristics of the sample expressed by means and standard deviations (*n* = 208).

Variables	Mean and SD
Age	48.45 ± 6.05
SPADI	76.97 ± 20.91
FAB	63.4 ± 14.6
FABm	63.3 ± 14.7
FABs	63.4 ± 14.6
FABe	63.9 ± 15

SPADI, shoulder pain and disability index; SD, standard deviation. FAB, fear-avoidance beliefs. FABm, moderate fear-avoidance beliefs. FABs, severe fear-avoidance beliefs. FABe, extreme fear-avoidance beliefs.

**Table 2:** Correlations between age, fear-avoidance beliefs, and shoulder pain intensity and disability (*n* = 208).

Variables	Age	SPADI Total Score	FACS Total Score
Age	1.00	0.02	0.02
SPADI total score	0.02	1.00	0.97
FACS total score	0.02	0.97 *	1.00

SPADI, shoulder pain and disability index; FACS, fear-avoidance component scale; * *p* < 0.05.

## Data Availability

Not applicable.
